# 贝伐珠单抗联合紫杉醇/卡铂治疗晚期非小细胞肺癌25例分析

**DOI:** 10.3779/j.issn.1009-3419.2012.01.02

**Published:** 2012-01-20

**Authors:** 肖 赵, 孟昭 王, 力 张, 龙芸 李, 巍 钟

**Affiliations:** 100730 北京，中国医学科学院北京协和医学院，北京协和医院呼吸内科 Department of Respiratory Diseases, Peking Union Medical College Hospital, Peking Union Medical College and Chinese Academy of Medical Sciences, Beijing 100730, China

**Keywords:** 肺肿瘤, 贝伐珠单抗, 不良反应, 治疗, Lung neoplasms, Bevacizumab, Adverse effect, Therapy

## Abstract

**背景与目的:**

SAiL（MO19390）研究是一项开放性、国际多中心、单组的临床试验，旨在评价一线使用贝伐珠单抗为基础的治疗在临床上的安全性及疗效。本文25例患者来自本研究中心入组SAiL试验的患者。

**方法:**

2007年8月-2008年2月在北京协和医院治疗的25例晚期非鳞非小细胞肺癌（non-squamous non-small cell lung cancer, NSNSCLC）患者，接受贝伐珠单抗联合紫杉醇/卡铂治疗，贝伐珠单抗剂量15 mg/kg。评价不良反应、客观有效率（objective response rate, ORR）、中位疾病进展时间（time to progression, TTP）和总生存期（overall survival, OS）。

**结果:**

最多见的不良反应为脱发、外周神经病变、皮疹、蛋白尿、恶心/呕吐、乏力、肌肉酸痛、鼻粘膜出血和高血压。17例（68%）评估为部分缓解（partial remission, PR），7例（28%）评估为疾病稳定（stable disease, SD），1例（4%）评估为疾病进展（progressive disease, PD），中位TTP为11.2个月，中位OS为19.3个月。

**结论:**

中国晚期NSNSCLC患者接受贝伐珠单抗治疗的耐受性好，可明显延长TTP和OS。

## Abstract (en)

**Background and objective:**

The current study reported the result of bevacizumab treatment administered to 25 Chinese patients with advanced non-small cell lung cancer (NSCLC) who were treated at the Peking Union Medical College Hospital as a part of the SAiL (MO19390) trial. This trial is an open, international multicenter, single-arm clinical study that assesses the safety and efficacy of first-line bevacizumab-based therapy in advanced NSCLC.

**Methods:**

Twenty-five Chinese patients with advanced non-squamous NSCLC received bevacizumab (15 mg/kg) combined with chemotherapy (carboplatin plus paclitaxel) treatment from August 2007 to February 2008. Adverse effects (AEs), objective response rate (ORR), median time to progression (TTP), and overall survival (OS) were measured.

**Results:**

AEs were generally mild and reversible. The most frequent AEs were alopecia, peripheral neuropathy, rash, proteinuria, nausea/vomitting, fatigue, myalgia, bleeding, and hypertension. The partial remission and stable disease rates were 68% and 28%, respectively. The median TTP and OS of all patients were 11.2 and 19.3 months, respectively.

**Conclusion:**

Bevacizumab combined with carboplatin-based chemotherapy may be well tolerated and beneficial for Chinese patients with non-squamous NSCLC.

贝伐珠单抗是针对血管内皮生长因子的单克隆抗体，通过抑制血管生成抑制肿瘤生长，对非鳞非小细胞肺癌（non-squamous non-small cell lung cancer, NSNSCLC）、结肠癌、乳腺癌和肾细胞癌等有较好疗效。国外研究^[1-4]^报道贝伐珠单抗联合紫杉醇和卡铂一线治疗NSNSCLC可以使患者的中位生存时间超过12个月。美国食品药品监督局已批准贝伐珠单抗联合紫杉醇和卡铂用于晚期NSNSCLC的一线治疗。但在国内贝伐珠单抗对于非小细胞肺癌并没有适应症，其在中国患者中的疗效和安全性都需进一步验证。SAiL（MO19390）研究是一项开放性、国际多中心、单组的Ⅳ期临床试验，主要目的在于评价一线使用贝伐珠单抗为基础的治疗在临床上的安全性^[5]^。本研究25例患者来自SAiL研究中心入组SAiL试验的患者。本文总结了北京协和医院使用贝伐珠单抗治疗NSCLC患者的安全性和疗效。

## 1 对象与方法

### 1.1 对象

所有患者均来自本研究中心2007年8月-2008年2月入组SAiL试验的患者。该研究在北京协和医院伦理委员会的监督下进行。

入组标准（参照SAiL试验）如下：≥18岁；签署书面知情同意书；均有细胞学和/或组织学证实为NSNSCLC（仅有痰细胞学者除外）；临床分期为Ⅲb期或Ⅳ期；ECOG PS评分为0分-1分；预期寿命≥12周。其它条件包括有充足的骨髓功能（中性粒细胞计数＞1.5×10^9^/L，血小板计数＞100×10^9^/L，血红蛋白＞10 g/L）；肝功能（总胆红素＜1.0倍正常上限，谷丙转氨酶＜1.5倍正常上限，谷草转氨酶＜1.5倍正常上限）；肾功能（血清肌苷水平＜1.5 mg/dL，尿素氮＜20 mg/dL）。

排除标准包括：鳞癌细胞为主的非小细胞和小细胞混合癌或腺鳞混合癌；有咳血史，定义为在入组前3个月内至少咳出鲜血2 mL/d；影像学检查显示有重要血管侵犯的证据或病灶有空洞形成；未控制的中枢神经系统转移；在入组前28天之内曾行大手术，或在研究期间预计需要进行大手术；在贝伐珠单抗滴注前24 h之内进行小手术；未治愈的伤口、活动性消化道溃疡或骨折；在入组前6个月之内曾有腹部瘘管、胃肠道穿孔或腹腔内脓肿病史；目前或近来（贝伐珠单抗第1次给药10天之内）采用阿司匹林（＞325 mg/d）或全剂量口服或肠外给予抗凝药；具有遗传性出血体质或凝血障碍的证据；未控制的高血压；需要在研究期间采用研究药物治疗的有临床意义的心脏病。

### 1.2 试验方法

患者接受紫杉醇联合卡铂化疗，紫杉醇剂量为175 mg/m^2^，第1天，卡铂AUC=6，第1天，21天为一个疗程。每周期第1天给予贝伐珠单抗15 mg/kg。贝伐珠单抗具体方案为溶解于100 mL生理盐水中，第1次输注90 min，第2次60 min，以后每次30 min。化疗最多6个疗程，如果化疗结束后疾病未出现进展，则继续使用贝伐珠单抗直至出现不能耐受的毒性、疾病进展或死亡时停用。

入组前21天内收集病史、体格检查、实验室检查（包括全血细胞计数、尿液分析、肝肾功能）、心电图、胸部CT、腹部B超、头部CT或MRI、骨核素扫描，作为基线值。21天为1个治疗周期，每6周评价疗效。评价疗效包括所有的阳性病灶。

本研究主要目的为评估贝伐珠单抗与紫杉醇+卡铂（PC）方案一线联合治疗NSNSCLC的安全性。记录所有的不良事件，根据NIH CTC 3.0标准分级。次要目的为评估疗效：客观有效率（objective response rate, ORR）、症状缓解情况、疾病进展时间（time to progression, TTP）和总生存期（overall survival, OS）。客观疗效评价根据RECIST标准。

### 1.3 统计方法

采用SPSS 16.0软件进行统计学分析。中位OS和中位TTP采用*Kaplan*-*Meier*方法计算。

## 2 结果

### 2.1 一般情况

本研究收集了2007年8月-2008年2月于北京协和医院入组SAiL试验的25例患者。平均年龄为61.6岁（43岁-72岁），＜60岁13例，≥60岁12例；平均体重为66.7 kg（43 kg-84 kg）；男14例，女11例；Ⅲb期7例，Ⅳ期18例；ECOG PS评分0分16例，1分9例；吸烟者11例，不吸烟者14例。患者临床特征及与SAiL试验整体基线人群的比较见表 1。

**1:** 患者临床特征及与SAiL试验总体人群的比较 Characteristics of the 25 patients and general population in SAiL trial

	Present study (*n*=25)	SAiL (*n*=2, 212)
Age (yr)	61.6	58.8
< 60	13 (52%)	-
≥60	12 (48%)	-
Gender		
Male	14 (56%)	1, 329 (60%)
Female	11 (44%)	883 (40%)
Stage		
Ⅲb	7 (28%)	436 (20%)
Ⅳ	18 (72%)	1, 775 (80%)
ECOG performance status		
0	16 (64%)	822 (37%)
1	9 (36%)	1, 251 (57%)
Smoking status^*^		
Never	14 (56%)	670 (30%)
Ever	11 (44%)	1, 542 (70%)
^*^Smoking statuses were based on records from the patients’first clinic visits and an ever smoker was defined as a person who had smoked greater than 100 cigarettes in a life time.		

### 2.2 不良反应

最常见的不良反应为脱发、外周神经病变、皮疹、蛋白尿、恶心/呕吐、乏力、肌肉酸痛、鼻粘膜出血和高血压。所有不良反应均为1级-3级，未见3级以上的严重不良反应。5例（20%）患者出现了达到3级的不良反应，其中脱发（4%）、外周神经病变（8%）、乏力（8%）、恶心/呕吐（4%）和肌肉酸痛（4%）。常见的不良反应见表 2。

**2:** 发生率大于3%的不良反应 Number (%) of patients who had an adverse event with incidence > 3%

	Grade 1	Grade 2	Grade 3	Total
Rash	11 (44%)	2 (8%)	0	13 (52%)
Alopecia	8 (32%)	13 (53%)	1 (4%)	23 (92%)
Peripheral neuropathy	10 (40%)	2 (8%)	2 (8%)	14 (56%)
Fatigue	3 (12%)	3 (12%)	2 (8%)	8 (32%)
Nausea/vomit	5 (20%)	5 (20%)	1 (4%)	11 (44%)
Myalgia	5 (20%)	3 (12%)	1 (4%)	9 (36%)
Bleeding	6 (24%)	2 (8%)	0	8 (32%)
Proteinuria	11 (44%)	2 (8%)	0	13 (52%)
Hyertension	0	3 (12%)	0	3 (12%)

其它少见的不良反应包括气胸2例，1例在合并化疗2个疗程后出现，疗效评价为部分缓解（partial remission, PR），经过保守治疗近1个月后好转，此后完成6个疗程化疗并在化疗结束后使用贝伐珠单抗达9个月；另外1例在完成化疗后单独使用贝伐珠单抗10个月后出现。咯血1例，为2个疗程后出现，少量咯血，疗效评价为疾病稳定（stable disease, SD），患者终止了贝伐珠单抗治疗，继续单用化疗。嗜酸性粒细胞升高1例，最高计数达到4×10^9^/L，使用强的松后降至正常。急性肾功能衰竭1例，化疗2个疗程后出现，疗效评价为疾病进展（progressive disease, PD），经过血液透析后肾功能恢复，此后改服吉非替尼。

### 2.3 疗效

25例患者的客观疗效中PR 17例（68%），SD 7例（28%），PD 1例（4%）。共21例疾病进展或死亡，中位TTP为11.2个月（95%CI: 10.4-12.0）；目前随访到16例患者的生存期，中位OS为19.3个月（95%CI: 13.2-25.5）（图 1）。

**1:**
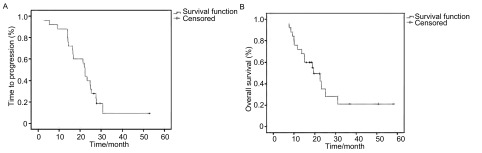
25例患者的疾病进展时间（A）和生存曲线（B） *Kaplan*-*Meier* estimates of time to progression (A) and overall survival (B) of the 25 patients

## 3 讨论

目前已有2项Ⅲ期随机对照临床试验（ECOG4599和AVAiL）报道贝伐珠单抗联合化疗一线治疗晚期或复发的NSNSCLC可以提高临床疗效^[2-4]^。ECOG 4599试验^[2]^一线使用贝伐珠单抗联合卡铂和紫杉醇，之后一直使用贝伐珠单抗直至疾病进展，与卡铂联合紫衫醇的标准化疗相比，中位TTP（6.2个月 *vs* 4.5个月）和中位OS（12.3个月 *vs* 10.3个月）均有所提高。在AVAiL试验^[3]^中，贝伐珠单抗一线联合卡铂和吉西他滨，贝伐珠单抗分为低剂量7.5 mg/kg组和高剂量15 mg/kg组。低剂量和高剂量贝伐珠单抗组与标准化疗组相比，中位TTP（6.7个月、6.5个月 *vs* 6.1个月）和中位OS（13.6个月、13.4个月 *vs* 13.1个月）均有所提高。基于上述2项Ⅲ期临床试验，SAiL试验的主要目标是评价在临床实践中贝伐珠单抗联合化疗一线治疗NSNSCLC的安全性，因此该实验没有规定统一的化疗方案，贝伐珠单抗剂量为15 mg/kg。本研究的25例患者均使用贝伐珠单抗联合紫杉醇和卡铂。

与之前的临床试验和SAiL的整体试验结果相比，本研究中患者的不良反应有自身特点（表 3），总的不良反应有所增加，包括脱发、皮疹、外周神经病变和蛋白尿，但严重不良反应低于之前报道的ECOG4599、AVAiL和SAiL临床试验。这3项试验报道最常见的严重不良反应为白细胞下降、胃肠道穿孔、出血和血栓事件等。上述常见的严重不良反应在本研究中仅有8例1级-2级出血，其余严重不良事件均未观察到，可能是本研究的例数较少造成的。

**3:** 试验中3/4度不良反应的比较 Grade 3/4 adverse events (%) in present and previous trials

	ECOG4599		AVAiL		SAiL		Present study
	PCB		GCB^*^		Chemo+B		PCB
Neutropenia	25.5		36		38		20
Thrombocytopenia	1.6		23		8		4
Anemia	0		10		-		0
Nausea/vomit	-		9		3		4
Myalgia	-		-		-		8
Peripheral neuropathy	-		-		0		8
Rash	2.3		-		-		0
Bleeding	4.4		4		4		0
Proteinuria	3.1		1		3		0
Hyertension	7		9		6		0
PCB: paclitaxel+carboplatin+bevacizumab; GCB: gemcitabine+carboplatin+bevacizumab; Chemo+B: chemotherapy+bevacizumab. ^*^ bevacizumab 15 mg/kg group.							

本研究中患者获得了较高的有效率（68%）、较长的TTP（11.2个月）和OS（19.3个月），总体疗效优于SAiL试验的整体疗效（ORR为52%，TTP为7.8个月，OS为14.6个月）。各项疗效指标均明显优于ECOG 4599报告的有效率35%、TTP 6.2个月和OS 12.3个月^[2]^，也高于Avail试验报告的30%、6.7个月和13.4个月^[3]^（表 4）。本研究中患者获得较好疗效的原因可能与亚洲人群表皮生长因子受体（epidermal growth factor receptor, *EGFR*）基因突变率较高有关^[6]^。IPASS试验^[6, 7]^显示*EGFR*基因突变的患者不管是应用标准化疗还是应用EGFR-TKIs治疗疗效和生存期均优于*EGFR*野生型患者。本研究的17例患者二、三线治疗使用了EGFR-TKIs，这也可能是生存期较长的原因之一。近期一项SAiL试验中亚洲（中国大陆、台湾和香港）人群的报道^[8]^显示贝伐珠单抗组的ORR、中位TTP和中位OS分别为56%、8.3个月和18.9个月，与本研究结果近似。该研究结果也证明了亚洲人群应用贝伐珠单抗可能会有更大的获益。

**4:** 本研究疗效与ECOG4599、AVAiL和SAiL试验的比较 Comparison of TTP, OS, ORR in ECOG4599, AVAiL, SAiL and present study

	ECOG4599 (month)	AVAiL^*^ (month)	SaiL (month)	Present Study (month)
TTP	6.2	6.5	7.8	11.2
OS	12.3	13.4	14.6	19.3
ORR	35%	30%	52%	68%
TTP: time to progression; OS: overall survival; ORR: objective response rate. ^*^ bevacizumab 15 mg/kg group.				

本研究的25例患者与SAiL试验的基线人群比较有如下特点（表 1）：PS评分为0分者比例较高（64% *vs* 37%），不吸烟者较多（56% *vs* 30%）。ECOG 4599和AVAiL试验中PS评分为0分者分别为40%和41%。本研究总不良反应少、严重不良反应少和疗效较好的原因，除了人种间差异和后续治疗的不同，也可能与PS评分较好有关。

总之，紫杉醇联合卡铂联合贝伐珠单抗一线治疗NSNSCLC的疗效有所提高，TTP和OS明显延长，而且不良反应耐受好，是有希望的联合治疗方案。
